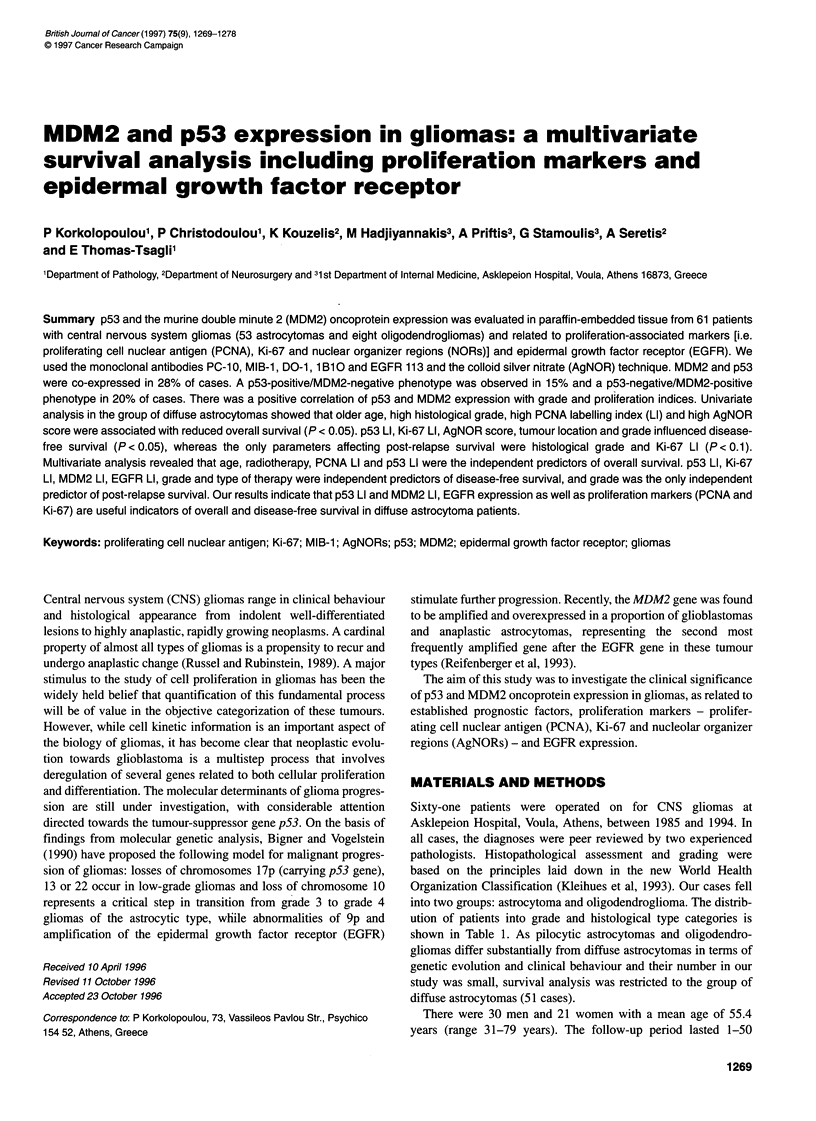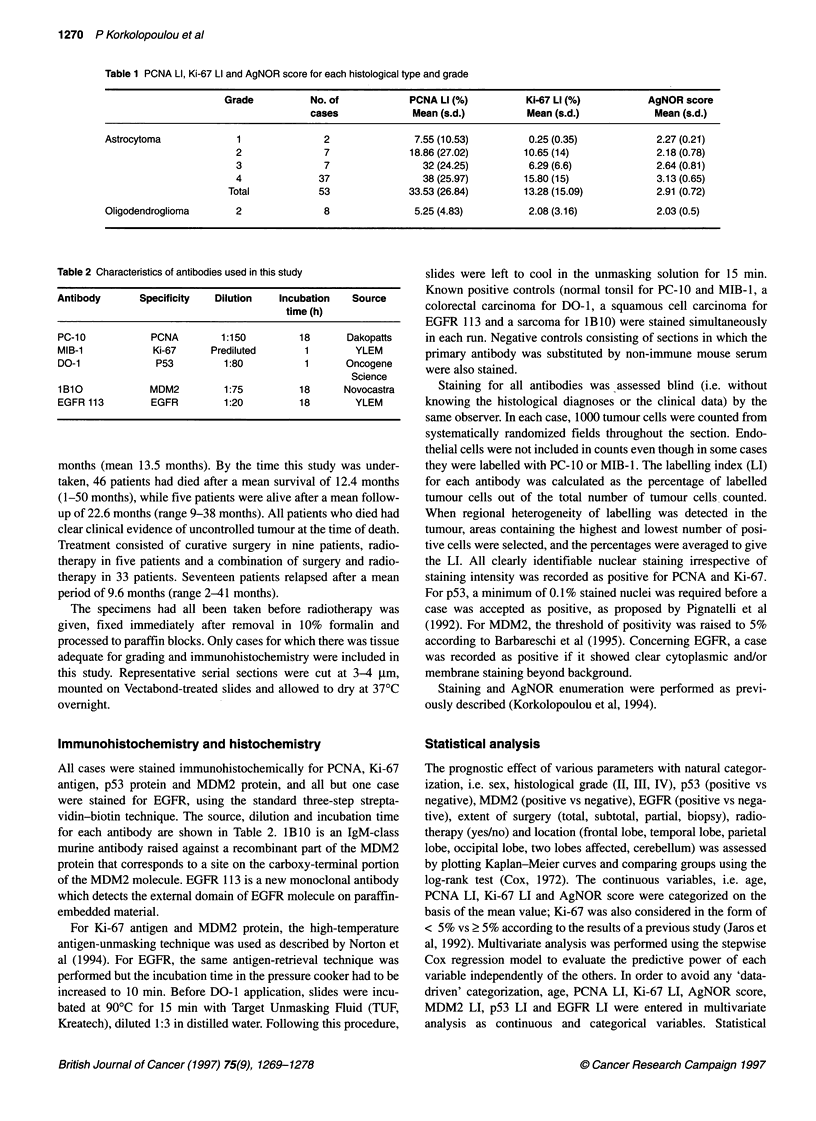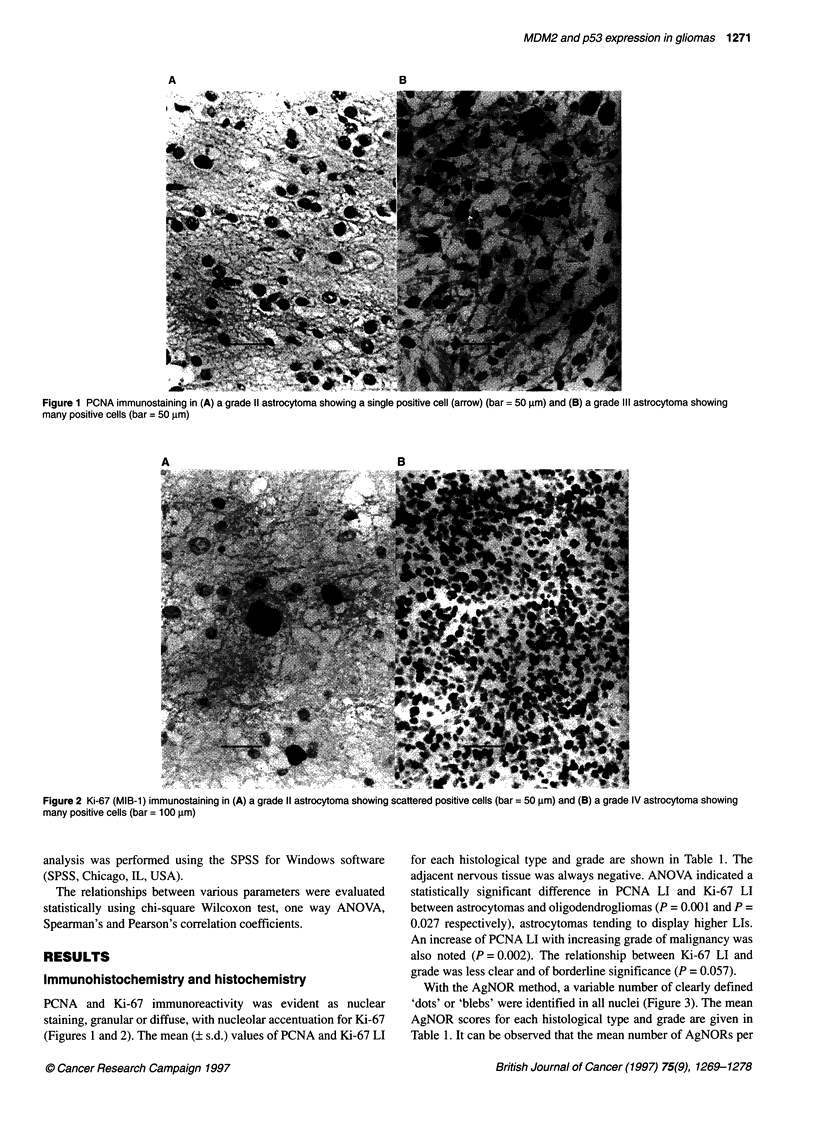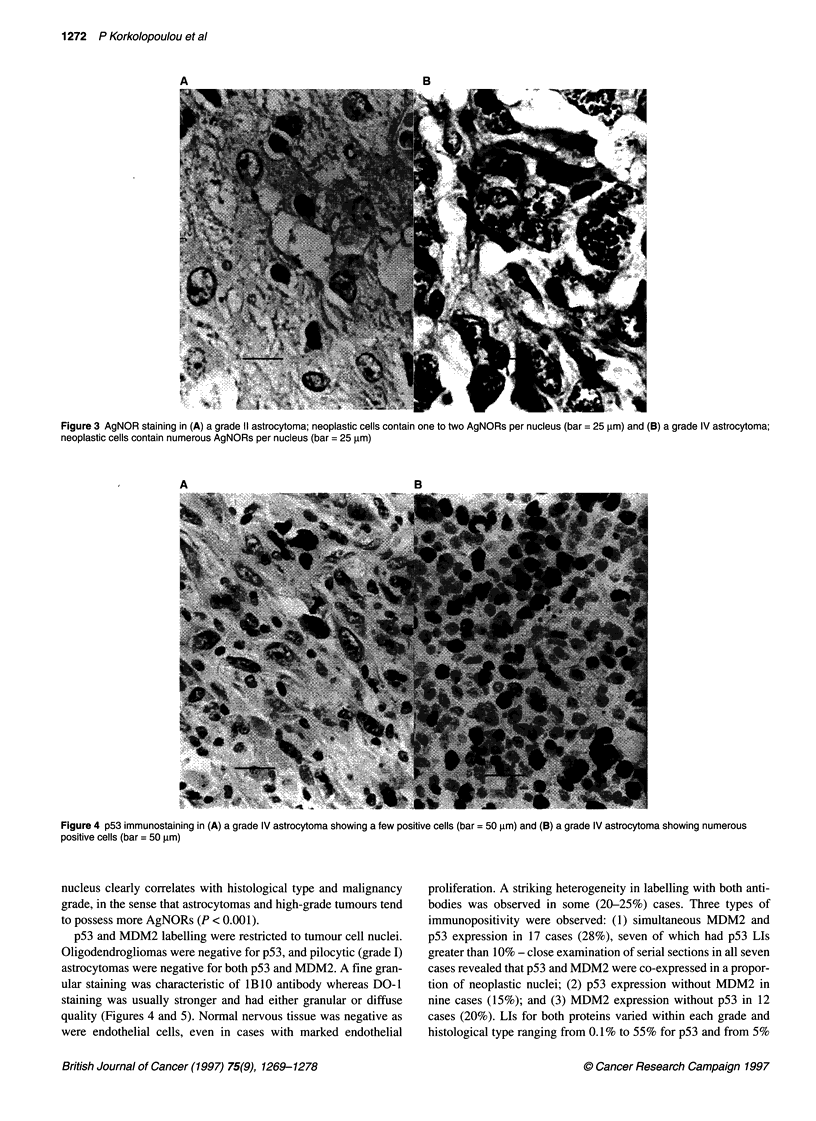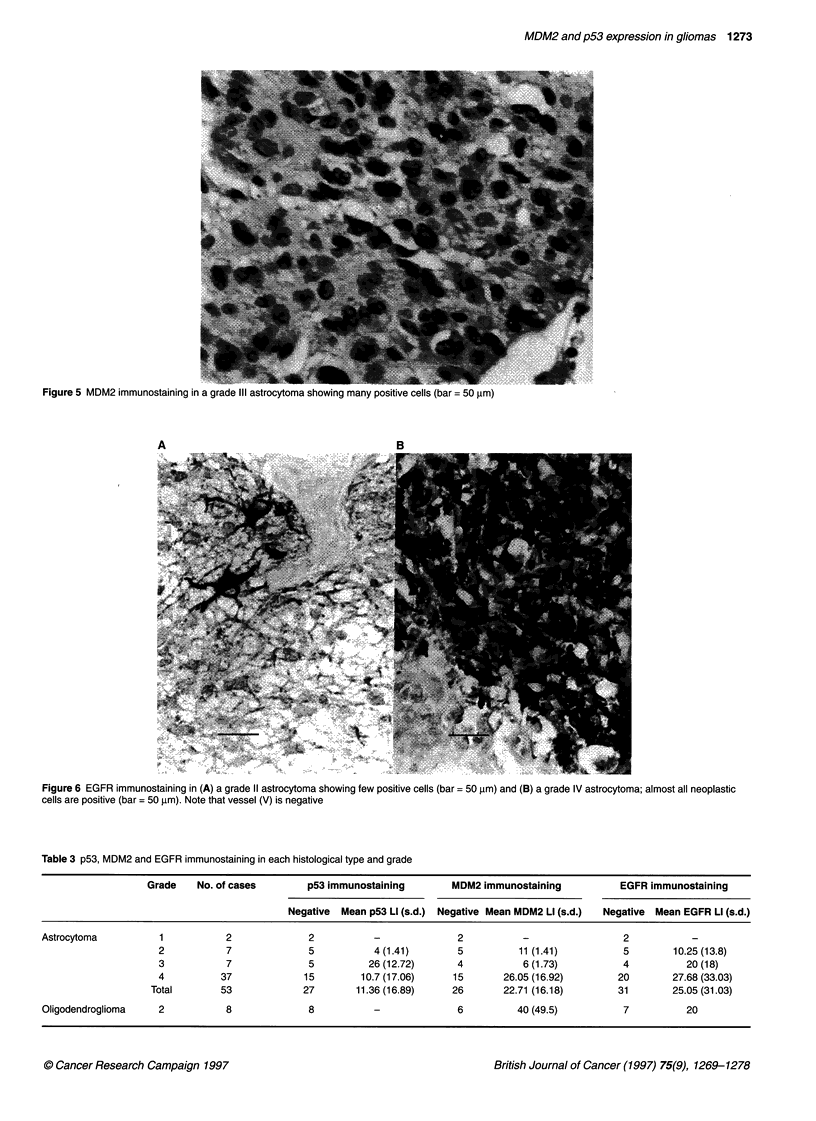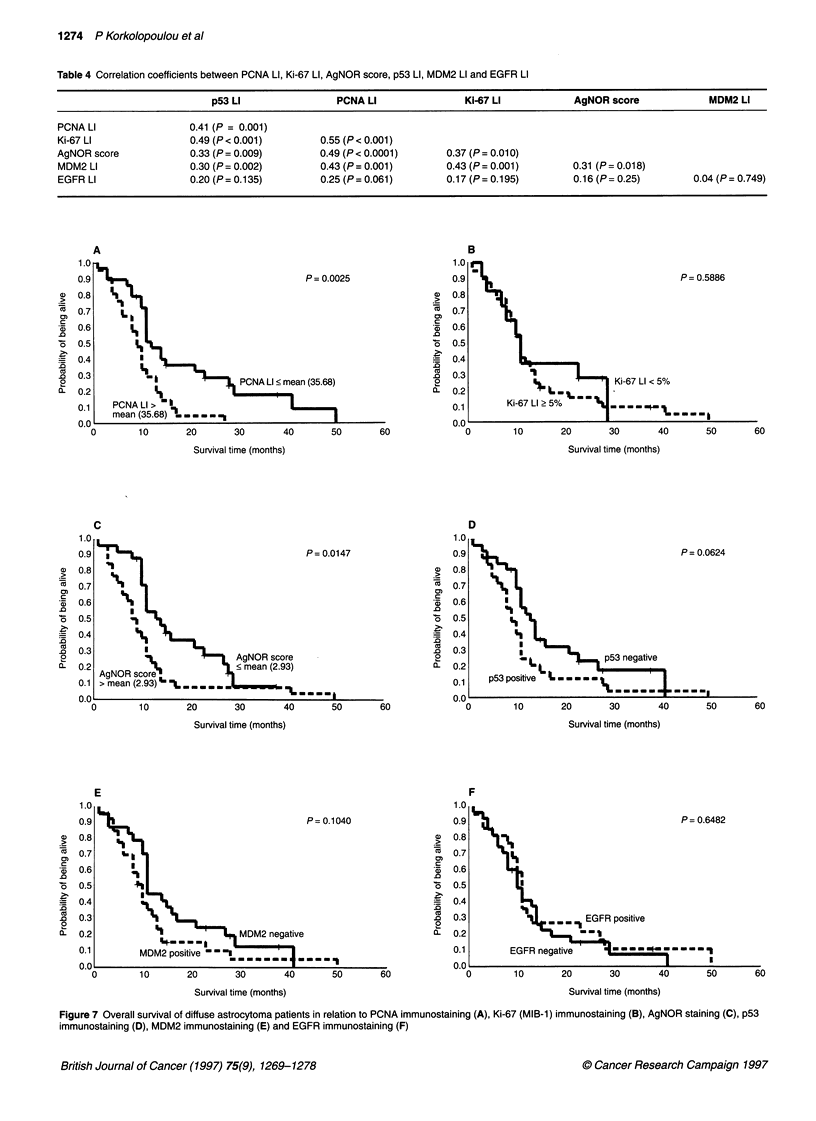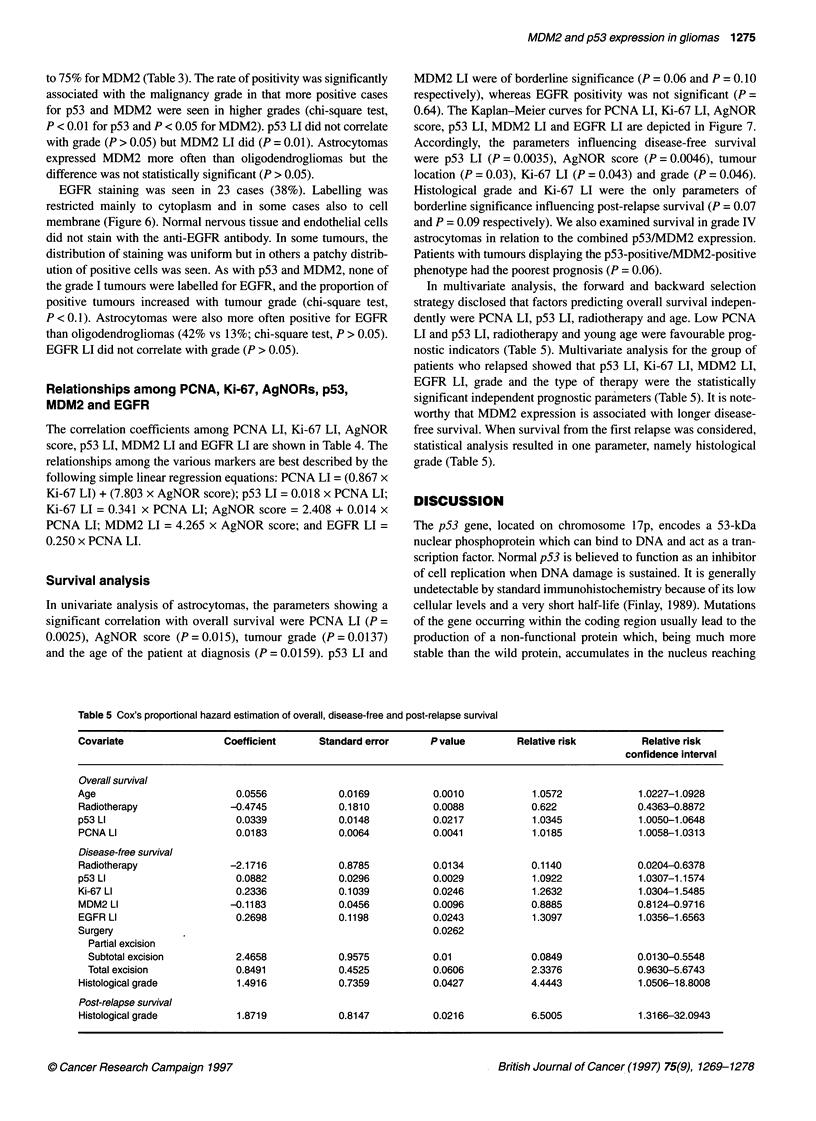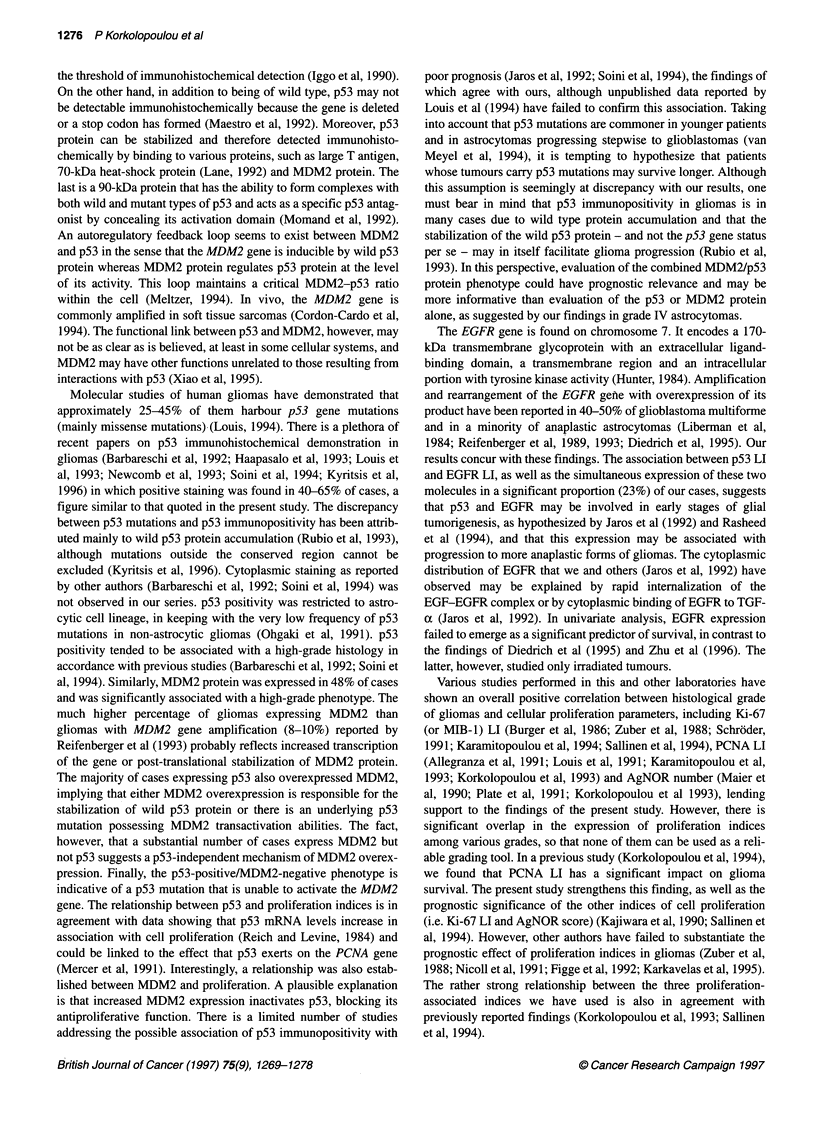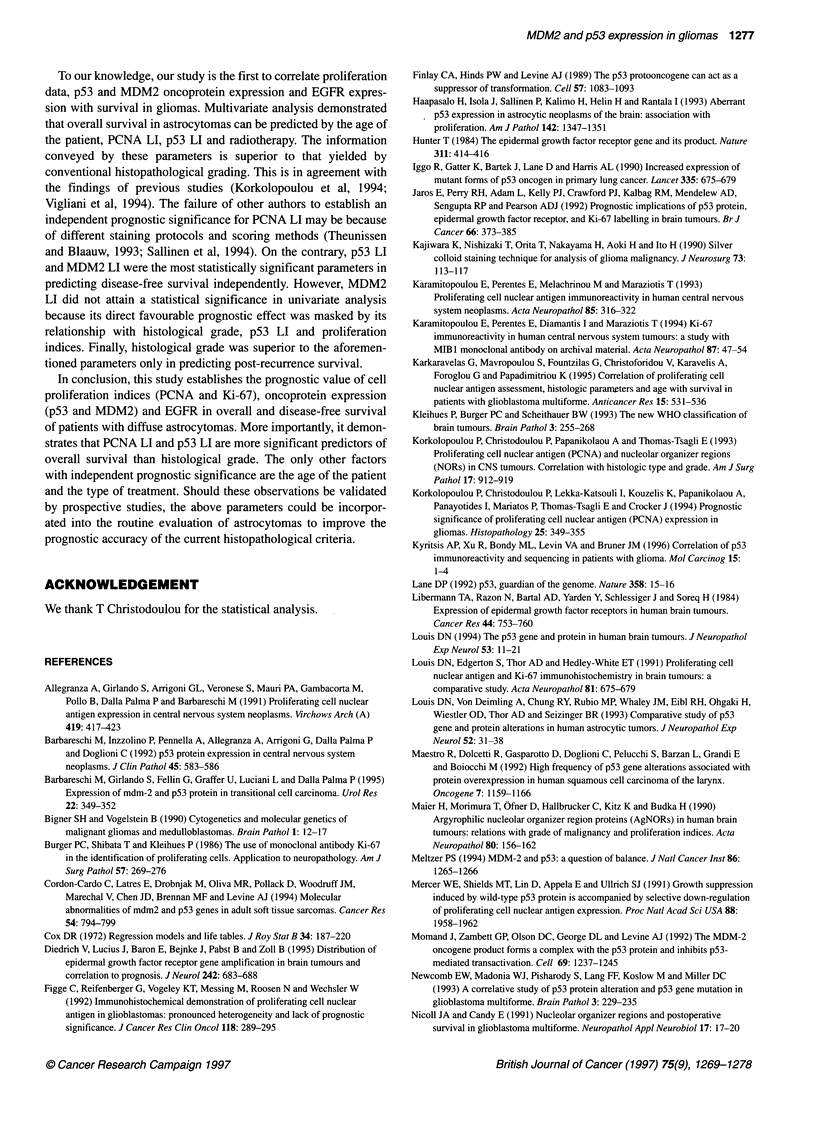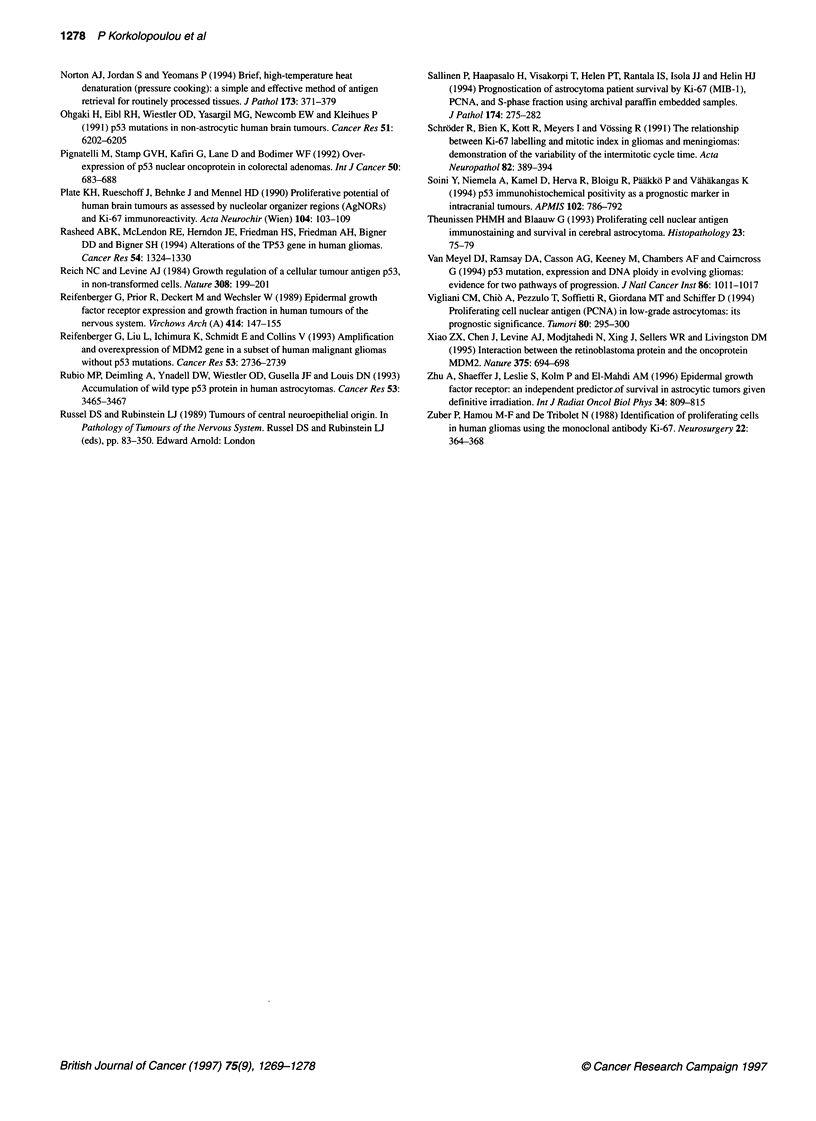# MDM2 and p53 expression in gliomas: a multivariate survival analysis including proliferation markers and epidermal growth factor receptor.

**DOI:** 10.1038/bjc.1997.216

**Published:** 1997

**Authors:** P. Korkolopoulou, P. Christodoulou, K. Kouzelis, M. Hadjiyannakis, A. Priftis, G. Stamoulis, A. Seretis, E. Thomas-Tsagli

**Affiliations:** Department of Pathology, Asklepeion Hospital, Voula, Athens, Greece.

## Abstract

**Images:**


British Journal of Cancer (1997) 75(9), 1269–1278
© 1997 Cancer Research Campaign

### MDM2 and p53 expression in gliomas: a multivariate survival analysis including proliferation markers and epidermal growth factor receptor

**P Korkolopoulou[1], P Christodoulou[1], K Kouzelis[2], M Hadjiyannakis[3], A Priftis[3], G Stamoulis[3], A Seretis[2] and E Thomas-Tsagli[1]**

[1]Department of Pathology, [2]Department of Neurosurgery and [3]1st Department of Internal Medicine, Asklepeion Hospital, Voula, Athens 16873, Greece

**Summary** p53 and the murine double minute 2 (MDM2) oncoprotein expression was evaluated in paraffin-embedded tissue from 61 patients with central nervous system gliomas (53 astrocytomas and eight oligodendrogliomas) and related to proliferation-associated markers [i.e. proliferating cell nuclear antigen (PCNA), Ki-67 and nuclear organizer regions (NORs)] and epidermal growth factor receptor (EGFR). We used the monoclonal antibodies PC-10, MIB-1, DO-1, 1B1O and EGFR 113 and the colloid silver nitrate (AgNOR) technique. MDM2 and p53 were co-expressed in 28% of cases. A p53-positive/MDM2-negative phenotype was observed in 15% and a p53-negative/MDM2-positive phenotype in 20% of cases. There was a positive correlation of p53 and MDM2 expression with grade and proliferation indices. Univariate analysis in the group of diffuse astrocytomas showed that older age, high histological grade, high PCNA labelling index (LI) and high AgNOR score were associated with reduced overall survival ($P < 0.05$). p53 LI, Ki-67 LI, AgNOR score, tumour location and grade influenced disease-free survival ($P < 0.05$), whereas the only parameters affecting post-relapse survival were histological grade and Ki-67 LI ($P < 0.1$). Multivariate analysis revealed that age, radiotherapy, PCNA LI and p53 LI were the independent predictors of overall survival. p53 LI, Ki-67 LI, MDM2 LI, EGFR LI, grade and type of therapy were independent predictors of disease-free survival, and grade was the only independent predictor of post-relapse survival. Our results indicate that p53 LI and MDM2 LI, EGFR expression as well as proliferation markers (PCNA and Ki-67) are useful indicators of overall and disease-free survival in diffuse astrocytoma patients.

**Keywords:** proliferating cell nuclear antigen; Ki-67; MIB-1; AgNORs; p53; MDM2; epidermal growth factor receptor; gliomas

Central nervous system (CNS) gliomas range in clinical behaviour and histological appearance from indolent well-differentiated lesions to highly anaplastic, rapidly growing neoplasms. A cardinal property of almost all types of gliomas is a propensity to recur and undergo anaplastic change (Russel and Rubinstein, 1989). A major stimulus to the study of cell proliferation in gliomas has been the widely held belief that quantification of this fundamental process will be of value in the objective categorization of these tumours. However, while cell kinetic information is an important aspect of the biology of gliomas, it has become clear that neoplastic evolution towards glioblastoma is a multistep process that involves deregulation of several genes related to both cellular proliferation and differentiation. The molecular determinants of glioma progression are still under investigation, with considerable attention directed towards the tumour-suppressor gene *p53*. On the basis of findings from molecular genetic analysis, Bigner and Vogelstein (1990) have proposed the following model for malignant progression of gliomas: losses of chromosomes 17p (carrying *p53* gene), 13 or 22 occur in low-grade gliomas and loss of chromosome 10 represents a critical step in transition from grade 3 to grade 4 gliomas of the astrocytic type, while abnormalities of 9p and amplification of the epidermal growth factor receptor (EGFR) stimulate further progression. Recently, the *MDM2* gene was found to be amplified and overexpressed in a proportion of glioblastomas and anaplastic astrocytomas, representing the second most frequently amplified gene after the EGFR gene in these tumour types (Reifenberger et al, 1993).

The aim of this study was to investigate the clinical significance of p53 and MDM2 oncoprotein expression in gliomas, as related to established prognostic factors, proliferation markers – proliferating cell nuclear antigen (PCNA), Ki-67 and nucleolar organizer regions (AgNORs) – and EGFR expression.

### MATERIALS AND METHODS

Sixty-one patients were operated on for CNS gliomas at Asklepeion Hospital, Voula, Athens, between 1985 and 1994. In all cases, the diagnoses were peer reviewed by two experienced pathologists. Histopathological assessment and grading were based on the principles laid down in the new World Health Organization Classification (Kleihues et al, 1993). Our cases fell into two groups: astrocytoma and oligodendroglioma. The distribution of patients into grade and histological type categories is shown in Table 1. As pilocytic astrocytomas and oligodendrogliomas differ substantially from diffuse astrocytomas in terms of genetic evolution and clinical behaviour and their number in our study was small, survival analysis was restricted to the group of diffuse astrocytomas (51 cases).

There were 30 men and 21 women with a mean age of 55.4 years (range 31–79 years). The follow-up period lasted 1–50

*Received 10 April 1996*
*Revised 11 October 1996*
*Accepted 23 October 1996*

*Correspondence to*: P Korkolopoulou, 73, Vassileos Pavlou Str., Psychico 154 52, Athens, Greece

**Table 1** PCNA LI, Ki-67 LI and AgNOR score for each histological type and grade

| | Grade | No. of cases | PCNA LI (%) Mean (s.d.) | Ki-67 LI (%) Mean (s.d.) | AgNOR score Mean (s.d.) |
|---|---|---|---|---|---|
| Astrocytoma | 1 | 2 | 7.55 (10.53) | 0.25 (0.35) | 2.27 (0.21) |
| | 2 | 7 | 18.86 (27.02) | 10.65 (14) | 2.18 (0.78) |
| | 3 | 7 | 32 (24.25) | 6.29 (6.6) | 2.64 (0.81) |
| | 4 | 37 | 38 (25.97) | 15.80 (15) | 3.13 (0.65) |
| | Total | 53 | 33.53 (26.84) | 13.28 (15.09) | 2.91 (0.72) |
| Oligodendroglioma | 2 | 8 | 5.25 (4.83) | 2.08 (3.16) | 2.03 (0.5) |

**Table 2** Characteristics of antibodies used in this study

| Antibody | Specificity | Dilution | Incubation time (h) | Source |
|---|---|---|---|---|
| PC-10 | PCNA | 1:150 | 18 | Dakopatts |
| MIB-1 | Ki-67 | Prediluted | 1 | YLEM |
| DO-1 | P53 | 1:80 | 1 | Oncogene Science |
| 1B1O | MDM2 | 1:75 | 18 | Novocastra |
| EGFR 113 | EGFR | 1:20 | 18 | YLEM |

months (mean 13.5 months). By the time this study was undertaken, 46 patients had died after a mean survival of 12.4 months (1–50 months), while five patients were alive after a mean follow-up of 22.6 months (range 9–38 months). All patients who died had clear clinical evidence of uncontrolled tumour at the time of death. Treatment consisted of curative surgery in nine patients, radiotherapy in five patients and a combination of surgery and radiotherapy in 33 patients. Seventeen patients relapsed after a mean period of 9.6 months (range 2–41 months).

The specimens had all been taken before radiotherapy was given, fixed immediately after removal in 10% formalin and processed to paraffin blocks. Only cases for which there was tissue adequate for grading and immunohistochemistry were included in this study. Representative serial sections were cut at 3–4 μm, mounted on Vectabond-treated slides and allowed to dry at 37°C overnight.

### Immunohistochemistry and histochemistry

All cases were stained immunohistochemically for PCNA, Ki-67 antigen, p53 protein and MDM2 protein, and all but one case were stained for EGFR, using the standard three-step streptavidin–biotin technique. The source, dilution and incubation time for each antibody are shown in Table 2. 1B10 is an IgM-class murine antibody raised against a recombinant part of the MDM2 protein that corresponds to a site on the carboxy-terminal portion of the MDM2 molecule. EGFR 113 is a new monoclonal antibody which detects the external domain of EGFR molecule on paraffin-embedded material.

For Ki-67 antigen and MDM2 protein, the high-temperature antigen-unmasking technique was used as described by Norton et al (1994). For EGFR, the same antigen-retrieval technique was performed but the incubation time in the pressure cooker had to be increased to 10 min. Before DO-1 application, slides were incubated at 90°C for 15 min with Target Unmasking Fluid (TUF, Kreatech), diluted 1:3 in distilled water. Following this procedure, slides were left to cool in the unmasking solution for 15 min. Known positive controls (normal tonsil for PC-10 and MIB-1, a colorectal carcinoma for DO-1, a squamous cell carcinoma for EGFR 113 and a sarcoma for 1B10) were stained simultaneously in each run. Negative controls consisting of sections in which the primary antibody was substituted by non-immune mouse serum were also stained.

Staining for all antibodies was assessed blind (i.e. without knowing the histological diagnoses or the clinical data) by the same observer. In each case, 1000 tumour cells were counted from systematically randomized fields throughout the section. Endothelial cells were not included in counts even though in some cases they were labelled with PC-10 or MIB-1. The labelling index (LI) for each antibody was calculated as the percentage of labelled tumour cells out of the total number of tumour cells counted. When regional heterogeneity of labelling was detected in the tumour, areas containing the highest and lowest number of positive cells were selected, and the percentages were averaged to give the LI. All clearly identifiable nuclear staining irrespective of staining intensity was recorded as positive for PCNA and Ki-67. For p53, a minimum of 0.1% stained nuclei was required before a case was accepted as positive, as proposed by Pignatelli et al (1992). For MDM2, the threshold of positivity was raised to 5% according to Barbareschi et al (1995). Concerning EGFR, a case was recorded as positive if it showed clear cytoplasmic and/or membrane staining beyond background.

Staining and AgNOR enumeration were performed as previously described (Korkolopoulou et al, 1994).

### Statistical analysis

The prognostic effect of various parameters with natural categorization, i.e. sex, histological grade (II, III, IV), p53 (positive vs negative), MDM2 (positive vs negative), EGFR (positive vs negative), extent of surgery (total, subtotal, partial, biopsy), radiotherapy (yes/no) and location (frontal lobe, temporal lobe, parietal lobe, occipital lobe, two lobes affected, cerebellum) was assessed by plotting Kaplan–Meier curves and comparing groups using the log-rank test (Cox, 1972). The continuous variables, i.e. age, PCNA LI, Ki-67 LI and AgNOR score were categorized on the basis of the mean value; Ki-67 was also considered in the form of $< 5\%$ vs $\geq 5\%$ according to the results of a previous study (Jaros et al, 1992). Multivariate analysis was performed using the stepwise Cox regression model to evaluate the predictive power of each variable independently of the others. In order to avoid any 'data-driven' categorization, age, PCNA LI, Ki-67 LI, AgNOR score, MDM2 LI, p53 LI and EGFR LI were entered in multivariate analysis as continuous and categorical variables. Statistical

 © Cancer Research Campaign 1997

**Figure 1** PCNA immunostaining in (**A**) a grade II astrocytoma showing a single positive cell (arrow) (bar = 50 μm) and (**B**) a grade III astrocytoma showing many positive cells (bar = 50 μm)

**Figure 2** Ki-67 (MIB-1) immunostaining in (**A**) a grade II astrocytoma showing scattered positive cells (bar = 50 μm) and (**B**) a grade IV astrocytoma showing many positive cells (bar = 100 μm)

analysis was performed using the SPSS for Windows software (SPSS, Chicago, IL, USA).

The relationships between various parameters were evaluated statistically using chi-square Wilcoxon test, one way ANOVA, Spearman's and Pearson's correlation coefficients.

### RESULTS

### Immunohistochemistry and histochemistry

PCNA and Ki-67 immunoreactivity was evident as nuclear staining, granular or diffuse, with nucleolar accentuation for Ki-67 (Figures 1 and 2). The mean (± s.d.) values of PCNA and Ki-67 LI for each histological type and grade are shown in Table 1. The adjacent nervous tissue was always negative. ANOVA indicated a statistically significant difference in PCNA LI and Ki-67 LI between astrocytomas and oligodendrogliomas ($P = 0.001$ and $P = 0.027$ respectively), astrocytomas tending to display higher LIs. An increase of PCNA LI with increasing grade of malignancy was also noted ($P = 0.002$). The relationship between Ki-67 LI and grade was less clear and of borderline significance ($P = 0.057$).

With the AgNOR method, a variable number of clearly defined 'dots' or 'blebs' were identified in all nuclei (Figure 3). The mean AgNOR scores for each histological type and grade are given in Table 1. It can be observed that the mean number of AgNORs per

© Cancer Research Campaign 1997

**Figure 3** AgNOR staining in (**A**) a grade II astrocytoma; neoplastic cells contain one to two AgNORs per nucleus (bar = 25 μm) and (**B**) a grade IV astrocytoma; neoplastic cells contain numerous AgNORs per nucleus (bar = 25 μm)

**Figure 4** p53 immunostaining in (**A**) a grade IV astrocytoma showing a few positive cells (bar = 50 μm) and (**B**) a grade IV astrocytoma showing numerous positive cells (bar = 50 μm)

nucleus clearly correlates with histological type and malignancy grade, in the sense that astrocytomas and high-grade tumours tend to possess more AgNORs ($P < 0.001$).

p53 and MDM2 labelling were restricted to tumour cell nuclei. Oligodendrogliomas were negative for p53, and pilocytic (grade I) astrocytomas were negative for both p53 and MDM2. A fine granular staining was characteristic of 1B10 antibody whereas DO-1 staining was usually stronger and had either granular or diffuse quality (Figures 4 and 5). Normal nervous tissue was negative as were endothelial cells, even in cases with marked endothelial proliferation. A striking heterogeneity in labelling with both antibodies was observed in some (20–25%) cases. Three types of immunopositivity were observed: (1) simultaneous MDM2 and p53 expression in 17 cases (28%), seven of which had p53 LIs greater than 10% – close examination of serial sections in all seven cases revealed that p53 and MDM2 were co-expressed in a proportion of neoplastic nuclei; (2) p53 expression without MDM2 in nine cases (15%); and (3) MDM2 expression without p53 in 12 cases (20%). LIs for both proteins varied within each grade and histological type ranging from 0.1% to 55% for p53 and from 5%

*© Cancer Research Campaign 1997*

**Figure 5** MDM2 immunostaining in a grade III astrocytoma showing many positive cells (bar = 50 μm)

**Figure 6** EGFR immunostaining in (**A**) a grade II astrocytoma showing few positive cells (bar = 50 μm) and (**B**) a grade IV astrocytoma; almost all neoplastic cells are positive (bar = 50 μm). Note that vessel (V) is negative

**Table 3** p53, MDM2 and EGFR immunostaining in each histological type and grade

| | Grade | No. of cases | p53 immunostaining | | MDM2 immunostaining | | EGFR immunostaining | |
|---|---|---|---|---|---|---|---|---|
| | | | Negative | Mean p53 LI (s.d.) | Negative | Mean MDM2 LI (s.d.) | Negative | Mean EGFR LI (s.d.) |
| Astrocytoma | 1 | 2 | 2 | – | 2 | – | 2 | – |
| | 2 | 7 | 5 | 4 (1.41) | 5 | 11 (1.41) | 5 | 10.25 (13.8) |
| | 3 | 7 | 5 | 26 (12.72) | 4 | 6 (1.73) | 4 | 20 (18) |
| | 4 | 37 | 15 | 10.7 (17.06) | 15 | 26.05 (16.92) | 20 | 27.68 (33.03) |
| | Total | 53 | 27 | 11.36 (16.89) | 26 | 22.71 (16.18) | 31 | 25.05 (31.03) |
| Oligodendroglioma | 2 | 8 | 8 | – | 6 | 40 (49.5) | 7 | 20 |

**Table 4** Correlation coefficients between PCNA LI, Ki-67 LI, AgNOR score, p53 LI, MDM2 LI and EGFR LI

| | p53 LI | PCNA LI | Ki-67 LI | AgNOR score | MDM2 LI |
|---|---|---|---|---|---|
| PCNA LI | 0.41 ($P$ = 0.001) | | | | |
| Ki-67 LI | 0.49 ($P$ < 0.001) | 0.55 ($P$ < 0.001) | | | |
| AgNOR score | 0.33 ($P$ = 0.009) | 0.49 ($P$ < 0.0001) | 0.37 ($P$ = 0.010) | | |
| MDM2 LI | 0.30 ($P$ = 0.002) | 0.43 ($P$ = 0.001) | 0.43 ($P$ = 0.001) | 0.31 ($P$ = 0.018) | |
| EGFR LI | 0.20 ($P$ = 0.135) | 0.25 ($P$ = 0.061) | 0.17 ($P$ = 0.195) | 0.16 ($P$ = 0.25) | 0.04 ($P$ = 0.749) |

**Figure 7** Overall survival of diffuse astrocytoma patients in relation to PCNA immunostaining (**A**), Ki-67 (MIB-1) immunostaining (**B**), AgNOR staining (**C**), p53 immunostaining (**D**), MDM2 immunostaining (**E**) and EGFR immunostaining (**F**)

© Cancer Research Campaign 1997

to 75% for MDM2 (Table 3). The rate of positivity was significantly associated with the malignancy grade in that more positive cases for p53 and MDM2 were seen in higher grades (chi-square test, $P < 0.01$ for p53 and $P < 0.05$ for MDM2). p53 LI did not correlate with grade ($P > 0.05$) but MDM2 LI did ($P = 0.01$). Astrocytomas expressed MDM2 more often than oligodendrogliomas but the difference was not statistically significant ($P > 0.05$).

EGFR staining was seen in 23 cases (38%). Labelling was restricted mainly to cytoplasm and in some cases also to cell membrane (Figure 6). Normal nervous tissue and endothelial cells did not stain with the anti-EGFR antibody. In some tumours, the distribution of staining was uniform but in others a patchy distribution of positive cells was seen. As with p53 and MDM2, none of the grade I tumours were labelled for EGFR, and the proportion of positive tumours increased with tumour grade (chi-square test, $P < 0.1$). Astrocytomas were also more often positive for EGFR than oligodendrogliomas (42% vs 13%; chi-square test, $P > 0.05$). EGFR LI did not correlate with grade ($P > 0.05$).

### Relationships among PCNA, Ki-67, AgNORs, p53, MDM2 and EGFR

The correlation coefficients among PCNA LI, Ki-67 LI, AgNOR score, p53 LI, MDM2 LI and EGFR LI are shown in Table 4. The relationships among the various markers are best described by the following simple linear regression equations: PCNA LI = (0.867 × Ki-67 LI) + (7.803 × AgNOR score); p53 LI = 0.018 × PCNA LI; Ki-67 LI = 0.341 × PCNA LI; AgNOR score = 2.408 + 0.014 × PCNA LI; MDM2 LI = 4.265 × AgNOR score; and EGFR LI = 0.250 × PCNA LI.

### Survival analysis

In univariate analysis of astrocytomas, the parameters showing a significant correlation with overall survival were PCNA LI ($P = 0.0025$), AgNOR score ($P = 0.015$), tumour grade ($P = 0.0137$) and the age of the patient at diagnosis ($P = 0.0159$). p53 LI and MDM2 LI were of borderline significance ($P = 0.06$ and $P = 0.10$ respectively), whereas EGFR positivity was not significant ($P = 0.64$). The Kaplan–Meier curves for PCNA LI, Ki-67 LI, AgNOR score, p53 LI, MDM2 LI and EGFR LI are depicted in Figure 7. Accordingly, the parameters influencing disease-free survival were p53 LI ($P = 0.0035$), AgNOR score ($P = 0.0046$), tumour location ($P = 0.03$), Ki-67 LI ($P = 0.043$) and grade ($P = 0.046$). Histological grade and Ki-67 LI were the only parameters of borderline significance influencing post-relapse survival ($P = 0.07$ and $P = 0.09$ respectively). We also examined survival in grade IV astrocytomas in relation to the combined p53/MDM2 expression. Patients with tumours displaying the p53-positive/MDM2-positive phenotype had the poorest prognosis ($P = 0.06$).

In multivariate analysis, the forward and backward selection strategy disclosed that factors predicting overall survival independently were PCNA LI, p53 LI, radiotherapy and age. Low PCNA LI and p53 LI, radiotherapy and young age were favourable prognostic indicators (Table 5). Multivariate analysis for the group of patients who relapsed showed that p53 LI, Ki-67 LI, MDM2 LI, EGFR LI, grade and the type of therapy were the statistically significant independent prognostic parameters (Table 5). It is noteworthy that MDM2 expression is associated with longer disease-free survival. When survival from the first relapse was considered, statistical analysis resulted in one parameter, namely histological grade (Table 5).

### DISCUSSION

The *p53* gene, located on chromosome 17p, encodes a 53-kDa nuclear phosphoprotein which can bind to DNA and act as a transcription factor. Normal *p53* is believed to function as an inhibitor of cell replication when DNA damage is sustained. It is generally undetectable by standard immunohistochemistry because of its low cellular levels and a very short half-life (Finlay, 1989). Mutations of the gene occurring within the coding region usually lead to the production of a non-functional protein which, being much more stable than the wild protein, accumulates in the nucleus reaching

**Table 5** Cox's proportional hazard estimation of overall, disease-free and post-relapse survival

| Covariate | Coefficient | Standard error | *P* value | Relative risk | Relative risk confidence interval |
|---|---|---|---|---|---|
| *Overall survival* | | | | | |
| Age | 0.0556 | 0.0169 | 0.0010 | 1.0572 | 1.0227–1.0928 |
| Radiotherapy | –0.4745 | 0.1810 | 0.0088 | 0.622 | 0.4363–0.8872 |
| p53 LI | 0.0339 | 0.0148 | 0.0217 | 1.0345 | 1.0050–1.0648 |
| PCNA LI | 0.0183 | 0.0064 | 0.0041 | 1.0185 | 1.0058–1.0313 |
| *Disease-free survival* | | | | | |
| Radiotherapy | –2.1716 | 0.8785 | 0.0134 | 0.1140 | 0.0204–0.6378 |
| p53 LI | 0.0882 | 0.0296 | 0.0029 | 1.0922 | 1.0307–1.1574 |
| Ki-67 LI | 0.2336 | 0.1039 | 0.0246 | 1.2632 | 1.0304–1.5485 |
| MDM2 LI | –0.1183 | 0.0456 | 0.0096 | 0.8885 | 0.8124–0.9716 |
| EGFR LI | 0.2698 | 0.1198 | 0.0243 | 1.3097 | 1.0356–1.6563 |
| Surgery | | | 0.0262 | | |
| Partial excision | | | | | |
| Subtotal excision | 2.4658 | 0.9575 | 0.01 | 0.0849 | 0.0130–0.5548 |
| Total excision | 0.8491 | 0.4525 | 0.0606 | 2.3376 | 0.9630–5.6743 |
| Histological grade | 1.4916 | 0.7359 | 0.0427 | 4.4443 | 1.0506–18.8008 |
| *Post-relapse survival* | | | | | |
| Histological grade | 1.8719 | 0.8147 | 0.0216 | 6.5005 | 1.3166–32.0943 |

© Cancer Research Campaign 1997

the threshold of immunohistochemical detection (Iggo et al, 1990). On the other hand, in addition to being of wild type, p53 may not be detectable immunohistochemically because the gene is deleted or a stop codon has formed (Maestro et al, 1992). Moreover, p53 protein can be stabilized and therefore detected immunohistochemically by binding to various proteins, such as large T antigen, 70-kDa heat-shock protein (Lane, 1992) and MDM2 protein. The last is a 90-kDa protein that has the ability to form complexes with both wild and mutant types of p53 and acts as a specific p53 antagonist by concealing its activation domain (Momand et al, 1992). An autoregulatory feedback loop seems to exist between MDM2 and p53 in the sense that the *MDM2* gene is inducible by wild p53 protein whereas MDM2 protein regulates p53 protein at the level of its activity. This loop maintains a critical MDM2–p53 ratio within the cell (Meltzer, 1994). In vivo, the *MDM2* gene is commonly amplified in soft tissue sarcomas (Cordon-Cardo et al, 1994). The functional link between p53 and MDM2, however, may not be as clear as is believed, at least in some cellular systems, and MDM2 may have other functions unrelated to those resulting from interactions with p53 (Xiao et al, 1995).

Molecular studies of human gliomas have demonstrated that approximately 25–45% of them harbour *p53* gene mutations (mainly missense mutations) (Louis, 1994). There is a plethora of recent papers on p53 immunohistochemical demonstration in gliomas (Barbareschi et al, 1992; Haapasalo et al, 1993; Louis et al, 1993; Newcomb et al, 1993; Soini et al, 1994; Kyritsis et al, 1996) in which positive staining was found in 40–65% of cases, a figure similar to that quoted in the present study. The discrepancy between p53 mutations and p53 immunopositivity has been attributed mainly to wild p53 protein accumulation (Rubio et al, 1993), although mutations outside the conserved region cannot be excluded (Kyritsis et al, 1996). Cytoplasmic staining as reported by other authors (Barbareschi et al, 1992; Soini et al, 1994) was not observed in our series. p53 positivity was restricted to astrocytic cell lineage, in keeping with the very low frequency of p53 mutations in non-astrocytic gliomas (Ohgaki et al, 1991). p53 positivity tended to be associated with a high-grade histology in accordance with previous studies (Barbareschi et al, 1992; Soini et al, 1994). Similarly, MDM2 protein was expressed in 48% of cases and was significantly associated with a high-grade phenotype. The much higher percentage of gliomas expressing MDM2 than gliomas with *MDM2* gene amplification (8–10%) reported by Reifenberger et al (1993) probably reflects increased transcription of the gene or post-translational stabilization of MDM2 protein. The majority of cases expressing p53 also overexpressed MDM2, implying that either MDM2 overexpression is responsible for the stabilization of wild p53 protein or there is an underlying p53 mutation possessing MDM2 transactivation abilities. The fact, however, that a substantial number of cases express MDM2 but not p53 suggests a p53-independent mechanism of MDM2 overexpression. Finally, the p53-positive/MDM2-negative phenotype is indicative of a p53 mutation that is unable to activate the *MDM2* gene. The relationship between p53 and proliferation indices is in agreement with data showing that p53 mRNA levels increase in association with cell proliferation (Reich and Levine, 1984) and could be linked to the effect that p53 exerts on the *PCNA* gene (Mercer et al, 1991). Interestingly, a relationship was also established between MDM2 and proliferation. A plausible explanation is that increased MDM2 expression inactivates p53, blocking its antiproliferative function. There is a limited number of studies addressing the possible association of p53 immunopositivity with poor prognosis (Jaros et al, 1992; Soini et al, 1994), the findings of which agree with ours, although unpublished data reported by Louis et al (1994) have failed to confirm this association. Taking into account that p53 mutations are commoner in younger patients and in astrocytomas progressing stepwise to glioblastomas (van Meyel et al, 1994), it is tempting to hypothesize that patients whose tumours carry p53 mutations may survive longer. Although this assumption is seemingly at discrepancy with our results, one must bear in mind that p53 immunopositivity in gliomas is in many cases due to wild type protein accumulation and that the stabilization of the wild p53 protein – and not the *p53* gene status per se – may in itself facilitate glioma progression (Rubio et al, 1993). In this perspective, evaluation of the combined MDM2/p53 protein phenotype could have prognostic relevance and may be more informative than evaluation of the p53 or MDM2 protein alone, as suggested by our findings in grade IV astrocytomas.

The *EGFR* gene is found on chromosome 7. It encodes a 170-kDa transmembrane glycoprotein with an extracellular ligand-binding domain, a transmembrane region and an intracellular portion with tyrosine kinase activity (Hunter, 1984). Amplification and rearrangement of the *EGFR* gene with overexpression of its product have been reported in 40–50% of glioblastoma multiforme and in a minority of anaplastic astrocytomas (Liberman et al, 1984; Reifenberger et al, 1989, 1993; Diedrich et al, 1995). Our results concur with these findings. The association between p53 LI and EGFR LI, as well as the simultaneous expression of these two molecules in a significant proportion (23%) of our cases, suggests that p53 and EGFR may be involved in early stages of glial tumorigenesis, as hypothesized by Jaros et al (1992) and Rasheed et al (1994), and that this expression may be associated with progression to more anaplastic forms of gliomas. The cytoplasmic distribution of EGFR that we and others (Jaros et al, 1992) have observed may be explained by rapid internalization of the EGF–EGFR complex or by cytoplasmic binding of EGFR to TGF-α (Jaros et al, 1992). In univariate analysis, EGFR expression failed to emerge as a significant predictor of survival, in contrast to the findings of Diedrich et al (1995) and Zhu et al (1996). The latter, however, studied only irradiated tumours.

Various studies performed in this and other laboratories have shown an overall positive correlation between histological grade of gliomas and cellular proliferation parameters, including Ki-67 (or MIB-1) LI (Burger et al, 1986; Zuber et al, 1988; Schröder, 1991; Karamitopoulou et al, 1994; Sallinen et al, 1994), PCNA LI (Allegranza et al, 1991; Louis et al, 1991; Karamitopoulou et al, 1993; Korkolopoulou et al, 1993) and AgNOR number (Maier et al, 1990; Plate et al, 1991; Korkolopoulou et al 1993), lending support to the findings of the present study. However, there is significant overlap in the expression of proliferation indices among various grades, so that none of them can be used as a reliable grading tool. In a previous study (Korkolopoulou et al, 1994), we found that PCNA LI has a significant impact on glioma survival. The present study strengthens this finding, as well as the prognostic significance of the other indices of cell proliferation (i.e. Ki-67 LI and AgNOR score) (Kajiwara et al, 1990; Sallinen et al, 1994). However, other authors have failed to substantiate the prognostic effect of proliferation indices in gliomas (Zuber et al, 1988; Nicoll et al, 1991; Figge et al, 1992; Karkavelas et al, 1995). The rather strong relationship between the three proliferation-associated indices we have used is also in agreement with previously reported findings (Korkolopoulou et al, 1993; Sallinen et al, 1994).

© Cancer Research Campaign 1997

To our knowledge, our study is the first to correlate proliferation data, p53 and MDM2 oncoprotein expression and EGFR expression with survival in gliomas. Multivariate analysis demonstrated that overall survival in astrocytomas can be predicted by the age of the patient, PCNA LI, p53 LI and radiotherapy. The information conveyed by these parameters is superior to that yielded by conventional histopathological grading. This is in agreement with the findings of previous studies (Korkolopoulou et al, 1994; Vigliani et al, 1994). The failure of other authors to establish an independent prognostic significance for PCNA LI may be because of different staining protocols and scoring methods (Theunissen and Blaauw, 1993; Sallinen et al, 1994). On the contrary, p53 LI and MDM2 LI were the most statistically significant parameters in predicting disease-free survival independently. However, MDM2 LI did not attain a statistical significance in univariate analysis because its direct favourable prognostic effect was masked by its relationship with histological grade, p53 LI and proliferation indices. Finally, histological grade was superior to the aforementioned parameters only in predicting post-recurrence survival.

In conclusion, this study establishes the prognostic value of cell proliferation indices (PCNA and Ki-67), oncoprotein expression (p53 and MDM2) and EGFR in overall and disease-free survival of patients with diffuse astrocytomas. More importantly, it demonstrates that PCNA LI and p53 LI are more significant predictors of overall survival than histological grade. The only other factors with independent prognostic significance are the age of the patient and the type of treatment. Should these observations be validated by prospective studies, the above parameters could be incorporated into the routine evaluation of astrocytomas to improve the prognostic accuracy of the current histopathological criteria.

### ACKNOWLEDGEMENT

We thank T Christodoulou for the statistical analysis.

### REFERENCES

Allegranza A, Girlando S, Arrigoni GL, Veronese S, Mauri PA, Gambacorta M, Pollo B, Dalla Palma P and Barbareschi M (1991) Proliferating cell nuclear antigen expression in central nervous system neoplasms. *Virchows Arch* (A) **419**: 417–423

Barbareschi M, Inzzolino P, Pennella A, Allegranza A, Arrigoni G, Dalla Palma P and Doglioni C (1992) p53 protein expression in central nervous system neoplasms. *J Clin Pathol* **45**: 583–586

Barbareschi M, Girlando S, Fellin G, Graffer U, Luciani L and Dalla Palma P (1995) Expression of mdm-2 and p53 protein in transitional cell carcinoma. *Urol Res* **22**: 349–352

Bigner SH and Vogelstein B (1990) Cytogenetics and molecular genetics of malignant gliomas and medulloblastomas. *Brain Pathol* **1**: 12–17

Burger PC, Shibata T and Kleihues P (1986) The use of monoclonal antibody Ki-67 in the identification of proliferating cells. Application to neuropathology. *Am J Surg Pathol* **57**: 269–276

Cordon-Cardo C, Latres E, Drobnjak M, Oliva MR, Pollack D, Woodruff JM, Marechal V, Chen JD, Brennan MF and Levine AJ (1994) Molecular abnormalities of mdm2 and p53 genes in adult soft tissue sarcomas. *Cancer Res* **54**: 794–799

Cox DR (1972) Regression models and life tables. *J Roy Stat B* **34**: 187–220

Diedrich V, Lucius J, Baron E, Bejnke J, Pabst B and Zoll B (1995) Distribution of epidermal growth factor receptor gene amplification in brain tumours and correlation to prognosis. *J Neurol* **242**: 683–688

Figge C, Reifenberger G, Vogeley KT, Messing M, Roosen N and Wechsler W (1992) Immunohistochemical demonstration of proliferating cell nuclear antigen in glioblastomas: pronounced heterogeneity and lack of prognostic significance. *J Cancer Res Clin Oncol* **118**: 289–295

Finlay CA, Hinds PW and Levine AJ (1989) The p53 protooncogene can act as a suppressor of transformation. *Cell* **57**: 1083–1093

Haapasalo H, Isola J, Sallinen P, Kalimo H, Helin H and Rantala I (1993) Aberrant p53 expression in astrocytic neoplasms of the brain: association with proliferation. *Am J Pathol* **142**: 1347–1351

Hunter T (1984) The epidermal growth factor receptor gene and its product. *Nature* **311**: 414–416

Iggo R, Gatter K, Bartek J, Lane D and Harris AL (1990) Increased expression of mutant forms of p53 oncogen in primary lung cancer. *Lancet* **335**: 675–679

Jaros E, Perry RH, Adam L, Kelly PJ, Crawford PJ, Kalbag RM, Mendelew AD, Sengupta RP and Pearson ADJ (1992) Prognostic implications of p53 protein, epidermal growth factor receptor, and Ki-67 labelling in brain tumours. *Br J Cancer* **66**: 373–385

Kajiwara K, Nishizaki T, Orita T, Nakayama H, Aoki H and Ito H (1990) Silver colloid staining technique for analysis of glioma malignancy. *J Neurosurg* **73**: 113–117

Karamitopoulou E, Perentes E, Melachrinou M and Maraziotis T (1993) Proliferating cell nuclear antigen immunoreactivity in human central nervous system neoplasms. *Acta Neuropathol* **85**: 316–322

Karamitopoulou E, Perentes E, Diamantis I and Maraziotis T (1994) Ki-67 immunoreactivity in human central nervous system tumours: a study with MIB1 monoclonal antibody on archival material. *Acta Neuropathol* **87**: 47–54

Karkaravelas G, Mavropoulou S, Fountzilas G, Christoforidou V, Karavelis A, Foroglou G and Papadimitriou K (1995) Correlation of proliferating cell nuclear antigen assessment, histologic parameters and age with survival in patients with glioblastoma multiforme. *Anticancer Res* **15**: 531–536

Kleihues P, Burger PC and Scheithauer BW (1993) The new WHO classification of brain tumours. *Brain Pathol* **3**: 255–268

Korkolopoulou P, Christodoulou P, Papanikolaou A and Thomas-Tsagli E (1993) Proliferating cell nuclear antigen (PCNA) and nucleolar organizer regions (NORs) in CNS tumours. Correlation with histologic type and grade. *Am J Surg Pathol* **17**: 912–919

Korkolopoulou P, Christodoulou P, Lekka-Katsouli I, Kouzelis K, Papanikolaou A, Panayotides I, Mariatos P, Thomas-Tsagli E and Crocker J (1994) Prognostic significance of proliferating cell nuclear antigen (PCNA) expression in gliomas. *Histopathology* **25**: 349–355

Kyritsis AP, Xu R, Bondy ML, Levin VA and Bruner JM (1996) Correlation of p53 immunoreactivity and sequencing in patients with glioma. *Mol Carcinog* **15**: 1–4

Lane DP (1992) p53, guardian of the genome. *Nature* **358**: 15–16

Libermann TA, Razon N, Bartal AD, Yarden Y, Schlessiger J and Soreq H (1984) Expression of epidermal growth factor receptors in human brain tumours. *Cancer Res* **44**: 753–760

Louis DN (1994) The p53 gene and protein in human brain tumours. *J Neuropathol Exp Neurol* **53**: 11–21

Louis DN, Edgerton S, Thor AD and Hedley-White ET (1991) Proliferating cell nuclear antigen and Ki-67 immunohistochemistry in brain tumours: a comparative study. *Acta Neuropathol* **81**: 675–679

Louis DN, Von Deimling A, Chung RY, Rubio MP, Whaley JM, Eibl RH, Ohgaki H, Wiestler OD, Thor AD and Seizinger BR (1993) Comparative study of p53 gene and protein alterations in human astrocytic tumors. *J Neuropathol Exp Neurol* **52**: 31–38

Maestro R, Dolcetti R, Gasparotto D, Doglioni C, Pelucchi S, Barzan L, Grandi E and Boiocchi M (1992) High frequency of p53 gene alterations associated with protein overexpression in human squamous cell carcinoma of the larynx. *Oncogene* **7**: 1159–1166

Maier H, Morimura T, Öfner D, Hallbrucker C, Kitz K and Budka H (1990) Argyrophilic nucleolar organizer region proteins (AgNORs) in human brain tumours: relations with grade of malignancy and proliferation indices. *Acta Neuropathol* **80**: 156–162

Meltzer PS (1994) MDM-2 and p53: a question of balance. *J Natl Cancer Inst* **86**: 1265–1266

Mercer WE, Shields MT, Lin D, Appela E and Ullrich SJ (1991) Growth suppression induced by wild-type p53 protein is accompanied by selective down-regulation of proliferating cell nuclear antigen expression. *Proc Natl Acad Sci USA* **88**: 1958–1962

Momand J, Zambett GP, Olson DC, George DL and Levine AJ (1992) The MDM-2 oncogene product forms a complex with the p53 protein and inhibits p53-mediated transactivation. *Cell* **69**: 1237–1245

Newcomb EW, Madonia WJ, Pisharody S, Lang FF, Koslow M and Miller DC (1993) A correlative study of p53 protein alteration and p53 gene mutation in glioblastoma multiforme. *Brain Pathol* **3**: 229–235

Nicoll JA and Candy E (1991) Nucleolar organizer regions and postoperative survival in glioblastoma multiforme. *Neuropathol Appl Neurobiol* **17**: 17–20

© Cancer Research Campaign 1997

Norton AJ, Jordan S and Yeomans P (1994) Brief, high-temperature heat denaturation (pressure cooking): a simple and effective method of antigen retrieval for routinely processed tissues. *J Pathol* **173**: 371–379

Ohgaki H, Eibl RH, Wiestler OD, Yasargil MG, Newcomb EW and Kleihues P (1991) p53 mutations in non-astrocytic human brain tumours. *Cancer Res* **51**: 6202–6205

Pignatelli M, Stamp GVH, Kafiri G, Lane D and Bodimer WF (1992) Over-expression of p53 nuclear oncoprotein in colorectal adenomas. *Int J Cancer* **50**: 683–688

Plate KH, Rueschoff J, Behnke J and Mennel HD (1990) Proliferative potential of human brain tumours as assessed by nucleolar organizer regions (AgNORs) and Ki-67 immunoreactivity. *Acta Neurochir* (Wien) **104**: 103–109

Rasheed ABK, McLendon RE, Herndon JE, Friedman HS, Friedman AH, Bigner DD and Bigner SH (1994) Alterations of the TP53 gene in human gliomas. *Cancer Res* **54**: 1324–1330

Reich NC and Levine AJ (1984) Growth regulation of a cellular tumour antigen p53, in non-transformed cells. *Nature* **308**: 199–201

Reifenberger G, Prior R, Deckert M and Wechsler W (1989) Epidermal growth factor receptor expression and growth fraction in human tumours of the nervous system. *Virchows Arch* (A) **414**: 147–155

Reifenberger G, Liu L, Ichimura K, Schmidt E and Collins V (1993) Amplification and overexpression of MDM2 gene in a subset of human malignant gliomas without p53 mutations. *Cancer Res* **53**: 2736–2739

Rubio MP, Deimling A, Ynadell DW, Wiestler OD, Gusella JF and Louis DN (1993) Accumulation of wild type p53 protein in human astrocytomas. *Cancer Res* **53**: 3465–3467

Russel DS and Rubinstein LJ (1989) Tumours of central neuroepithelial origin. In *Pathology of Tumours of the Nervous System.* Russel DS and Rubinstein LJ (eds), pp. 83–350. Edward Arnold: London

Sallinen P, Haapasalo H, Visakorpi T, Helen PT, Rantala IS, Isola JJ and Helin HJ (1994) Prognostication of astrocytoma patient survival by Ki-67 (MIB-1), PCNA, and S-phase fraction using archival paraffin embedded samples. *J Pathol* **174**: 275–282

Schröder R, Bien K, Kott R, Meyers I and Vössing R (1991) The relationship between Ki-67 labelling and mitotic index in gliomas and meningiomas: demonstration of the variability of the intermitotic cycle time. *Acta Neuropathol* **82**: 389–394

Soini Y, Niemela A, Kamel D, Herva R, Bloigu R, Pääkkö P and Vähäkangas K (1994) p53 immunohistochemical positivity as a prognostic marker in intracranial tumours. *APMIS* **102**: 786–792

Theunissen PHMH and Blaauw G (1993) Proliferating cell nuclear antigen immunostaining and survival in cerebral astrocytoma. *Histopathology* **23**: 75–79

Van Meyel DJ, Ramsay DA, Casson AG, Keeney M, Chambers AF and Cairncross G (1994) p53 mutation, expression and DNA ploidy in evolving gliomas: evidence for two pathways of progression. *J Natl Cancer Inst* **86**: 1011–1017

Vigliani CM, Chiò A, Pezzulo T, Soffietti R, Giordana MT and Schiffer D (1994) Proliferating cell nuclear antigen (PCNA) in low-grade astrocytomas: its prognostic significance. *Tumori* **80**: 295–300

Xiao ZX, Chen J, Levine AJ, Modjtahedi N, Xing J, Sellers WR and Livingston DM (1995) Interaction between the retinoblastoma protein and the oncoprotein MDM2. *Nature* **375**: 694–698

Zhu A, Shaeffer J, Leslie S, Kolm P and El-Mahdi AM (1996) Epidermal growth factor receptor: an independent predictor of survival in astrocytic tumors given definitive irradiation. *Int J Radiat Oncol Biol Phys* **34**: 809–815

Zuber P, Hamou M-F and De Tribolet N (1988) Identification of proliferating cells in human gliomas using the monoclonal antibody Ki-67. *Neurosurgery* **22**: 364–368

*© Cancer Research Campaign 1997*